# Supplementary material for: Specific Methyl-CpG Configurations Define Cell Identity through Gene Expression Regulation
Source: Int J Mol Sci. 2023 Jun 9;24(12):9951. doi: 10.3390/ijms24129951 (PMC10298370; doi:10.3390/ijms24129951)
Supplement: Supplementary file 1 [file ijms-24-09951-s001.zip › ijms-2428173-supplementary.pdf]

SUPPLEMENTARY INFORMATION

Specific Methyl-CpG Configurations Define Cell Identity through Gene Expression Regulation

Teresa Improda [1,†], Valentina Morgera [1,†], Maria Vitale [1], Lorenzo Chiariotti [2], Fabiana Passaro [2], Antonia Feola [1], Antonio Porcellini [1], Mariella Cuomo [2,*] and Antonio Pezone [1,*]

[1] Dipartimento di Biologia, Complesso Universitario di Monte Sant'Angelo, Università degli Studi di Napoli "Federico II", Napoli 80126, Italy

[2] Dipartimento di Medicina Molecolare e Biotecnologie Mediche, Università degli Studi di Napoli "Federico II", Napoli 80131, Italy

*Correspondence: mariella.cuomo@unina.it (M.C.); antonio.pezone@unina.it (A.Pezone)

†These authors equally contributed to this work.

Supplementary Figures

Figure S1. Both-strand methylation status on the BDNF promoter during stem cell differentiation.
(A) Average methylation of the six CpGs of the BDNF during stem cell differentiation and the relative random control at different time points (T0, T2, T4, T8 and T14, color-coded squares on the right). (B) Average methylation of each CpG identified by the nucleotide position. (C) Taxonomy plot of epialleles derived from each population. (D) Shannon entropy of the methylated molecules in the same samples and time points shown in (A). (E) Comparison of the Average methylation of each CpG identified by the nucleotide position shown in (B). (F) Pearson correlation of all analyzed time points. A pairwise comparison between each pair was performed with the student's t-test: *$P < 0.05$ versus T0.

Figure S2. Methylation signatures of BDNF promoters during stem cells differentiation.
(A) PCA of BDNF epialleles during stem cell differentiation and the relative random control at different time points (T0, T2, T4, T8 and T14, color-coded squares on the right). Dendrogram and hierarchical clustering of the frequency (B) and the structure (C) of BDNF epialleles. The similarity of different samples is represented by the horizontal distances on each branch of the dendrogram. 'vegan' R package (version 2.4-1) was used to perform the tests and draw correlograms. (D) Frequency of the methylated cores (MethCores index) in the same population analyzed in Fig. S1A. (E) Frequency of the methylated cores normalized to the average methylation (clonality index) in each population. (F) The structure and the composition of the methylated cores at different time points in the populations of molecules derived from each strand. Each CpG is labeled with a color code on the panel's right side. A pairwise comparison between each pair was performed with the student's t-test: *$P < 0.05$ versus T0.

Figure S3. DDO gene's methylation signatures during stem cell differentiation at region R1.
(A)Structure of the putative mouse *DDO* gene promoter. The blue arrow indicates the transcription start site (+1). Positions of CpG sites are indicated as relative to TSS. The analyzed 3 kb genomic portion is divided in 7 regions (R1-R2-R3-R4-R5-R6-R7) corresponding to different amplicons. (B) Average methylation of the 3 CpGs present in R1 shown in (A) and the relative random control at different time points (P0 and P30, color-coded squares on the right). (C) Average methylation of each CpG identified by the nucleotide position shown in (A), in the populations of molecules shown in (B). (D) Shannon entropy of the methylated molecules in the same samples and time points shown in (B). A pairwise comparison between each pair was performed with the student's t-test: *$P < 0.05$ versus P0. (E) The methylated cores' structure and composition at different times. Each CpG is labeled with a color code on the panel's right side.

Figure S4. DDO gene's methylation signatures during stem cell differentiation at regions R2 and R3.
(A)Average methylation of the 4 CpGs present in R2 shown in (Fig. S3A) and the relative random control at different time points (P0 and P30, color-coded squares on the right). (B) Average methylation of each CpG identified by the nucleotide position shown in (Fig. S3A), in the populations of molecules shown in (A). (C) (Shannon entropy of the methylated molecules in the same samples and time points shown in (A). (D) The methylated cores' structure and composition at different time points. Each CpG is labeled with a color code on the panel's right side. (E) Average methylation of the 9 CpGs present in R3 shown in (Fig. S3A) and the relative random control at different time points (P0 and P30, color-coded squares on the right). (F) Average methylation of each CpG identified by the nucleotide position shown in (Fig. S3A), in the populations of molecules shown in (E). (G) Shannon entropy of the methylated molecules in the same samples and time points shown in (E). (H) The methylated cores' structure and composition at different time points. Each CpG is labeled with a color code on the panel's right side. A pairwise comparison between each pair was performed with the student's t-test: *$P < 0.05$ versus P0.

Figure S5. DDO gene's methylation signatures during stem cell differentiation at regions R4 and R5.
(A)Average methylation of the 6 CpGs present in R4 shown in (Fig. S3A) and the relative random control at different time points (E15, P0, P14 and P30, color-coded squares on the right). (B) Average methylation of each CpG identified by the nucleotide position shown in (Fig. S3A), in the populations of molecules shown in (A). (C) (Shannon entropy of the methylated molecules in the same samples and time points shown in (A). (D) The methylated cores' structure and composition at different time points. Each CpG is labeled with a color code on the panel's right side. (E) Average methylation of the 6 CpGs present in R5 shown in (Fig. S3A) and the relative random control at different time points (E15, P0 and P30, color-coded squares on the right). (F) Average methylation of each CpG identified by the nucleotide position shown in (Fig. S3A), in the populations of molecules shown in (E). (G) Shannon entropy of the methylated molecules in the same samples and time points shown in (E). (H) The methylated cores' structure and composition at different time points. Each CpG is labeled with a color code on the panel's right side. A pairwise comparison between each pair was performed with the student's t-test: *$P < 0.05$ versus P0.

Figure S6. DDO gene's methylation signatures (cores) during stem cell differentiation at regions R6 and R7.
(A)Average methylation of the 6 CpGs present in R6 shown in (Fig. S3A) and the relative random control at different time points (E15, P0 and P30, color-coded squares on the right). (B) Average methylation of each CpG identified by the nucleotide position shown in (Fig. S3A), in the populations of molecules shown in (A). (C) (Shannon entropy of the methylated molecules in the same samples and time points shown in (A). (D) The methylated cores' structure and composition at different time points. Each CpG is labeled with a color code on the panel's right side. (E) Average methylation of the 9 CpGs present in R7 shown in (Fig. S3A) and the relative random control at different time points (P0 and P30, color-coded squares on the right). (F) Average methylation of each CpG identified by the nucleotide position shown in (Fig. S3A), in the populations of molecules shown in (E). (G) Shannon entropy of the methylated molecules in the same samples and time points shown in (E). (H) The methylated cores' structure and composition at different time points. Each CpG is labeled with a color code on the panel's right side. A pairwise comparison between each pair was performed with the student's t-test: *$P < 0.05$ versus P0.

Figure S7. The methylation status of DDO promoter in different tissues and cell lines. (A) The average methylation of the six CpGs shown in (Fig. S3A) of in the DNA extracted from brain areas, several cell types isolated from the same brain areas, immortalized A1 (neuronal) cells, c-myc immortalized neurons at different passages and ESCs upon induction of neural differentiation and the relative random control (B) Average methylation of each CpG identified by the nucleotide position shown in (Fig. S3A) in the populations of molecules shown in (A). (C) Shannon entropy of molecules in the same samples and time points shown in (A). (D) The methylated cores' structure and composition at different time points. Each CpG is labeled with a color code on the panel's right side. A pairwise comparison between each pair was performed with the student's t-test: *$P < 0.05$ versus T0.

Fig. S1

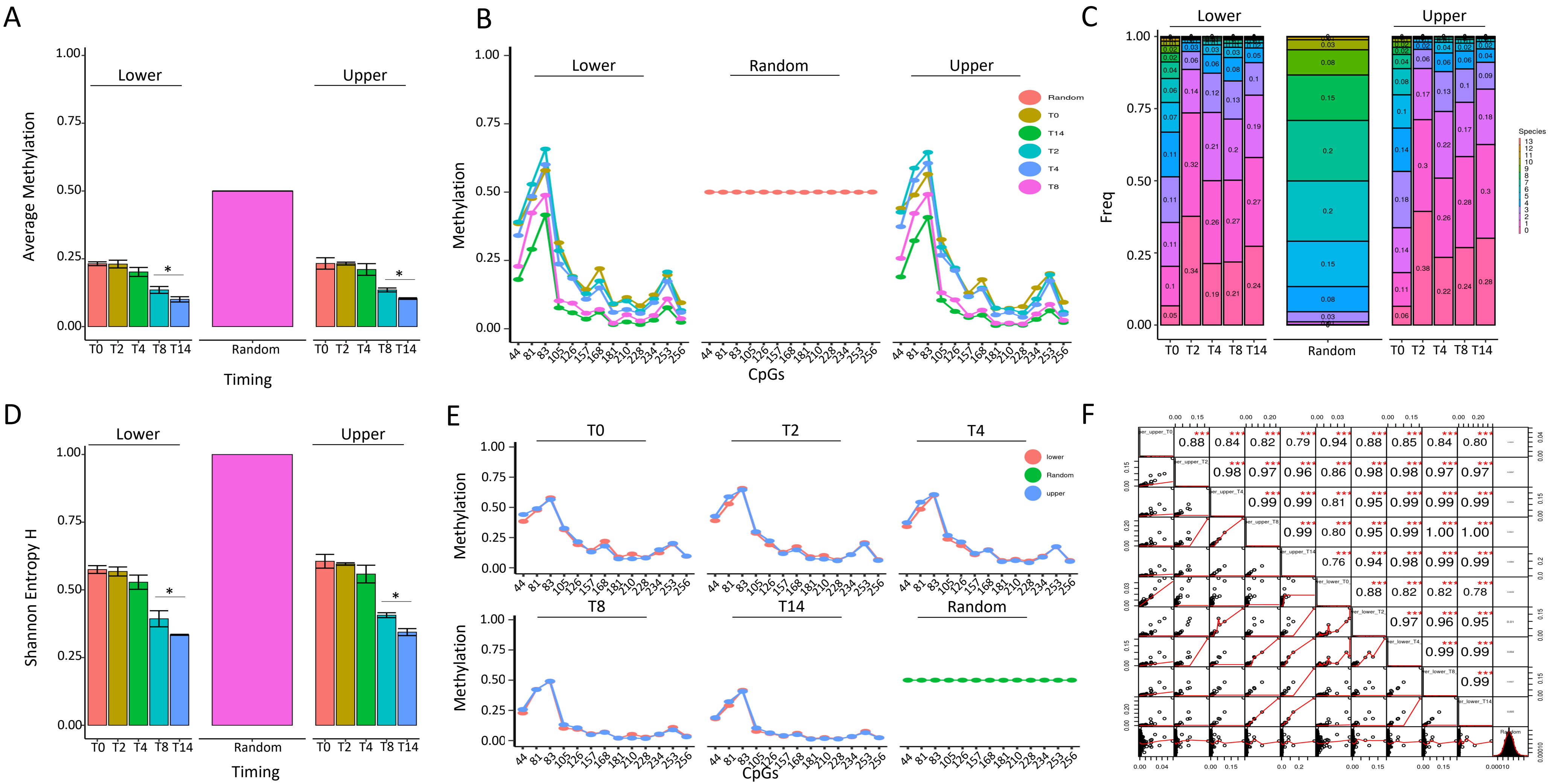

A
Average Methylation
Lower
Upper
Random
T0 T2 T4 T8 T14
Timing
B
Methylation
Lower
Random
Upper
CpGs
C
Freq
Lower
Random
Upper
Species
D
Shannon Entropy H
Lower
Upper
Random
Timing
E
Methylation
T0
T2
T4
T8
T14
Random
lower
Random
upper
CpGs
F

Fig. S2

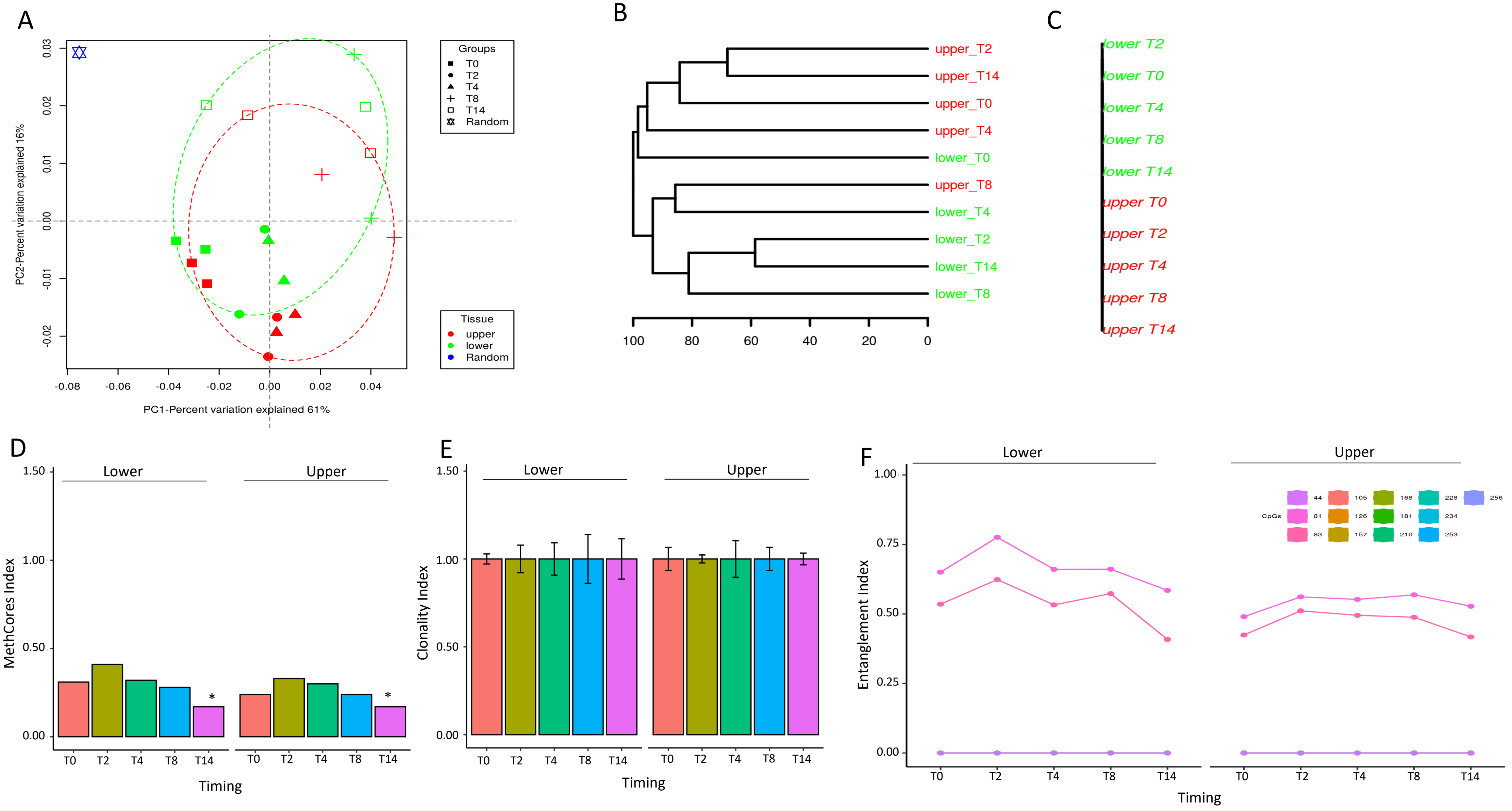
A
Groups
T0
T2
T4
T8
T14
Random
Tissue
upper
lower
Random
PC2-Percent variation explained 16%
PC1-Percent variation explained 61%
0.03
0.02
0.01
0.00
-0.01
-0.02
-0.08
-0.06
-0.04
-0.02
0.00
0.02
0.04
B
upper_T2
upper_T14
upper_T0
upper_T4
lower_T0
upper_T8
lower_T4
lower_T2
lower_T14
lower_T8
100
80
60
40
20
0
C
lower T2
lower T0
lower T4
lower T8
lower T14
upper T0
upper T2
upper T4
upper T8
upper T14
D
Lower
Upper
MethCores Index
1.50
1.00
0.50
0.00
*
*
T0 T2 T4 T8 T14 T0 T2 T4 T8 T14
Timing
E
Lower
Upper
Clonality Index
1.50
1.00
0.50
0.00
T0 T2 T4 T8 T14 T0 T2 T4 T8 T14
Timing
F
Lower
Upper
Entanglement Index
1.00
0.75
0.50
0.25
0.00
CpGs
44 105 168 228 256
81 126 181 234
83 157 210 253
T0 T2 T4 T8 T14 T0 T2 T4 T8 T14
Timing

Fig. S3

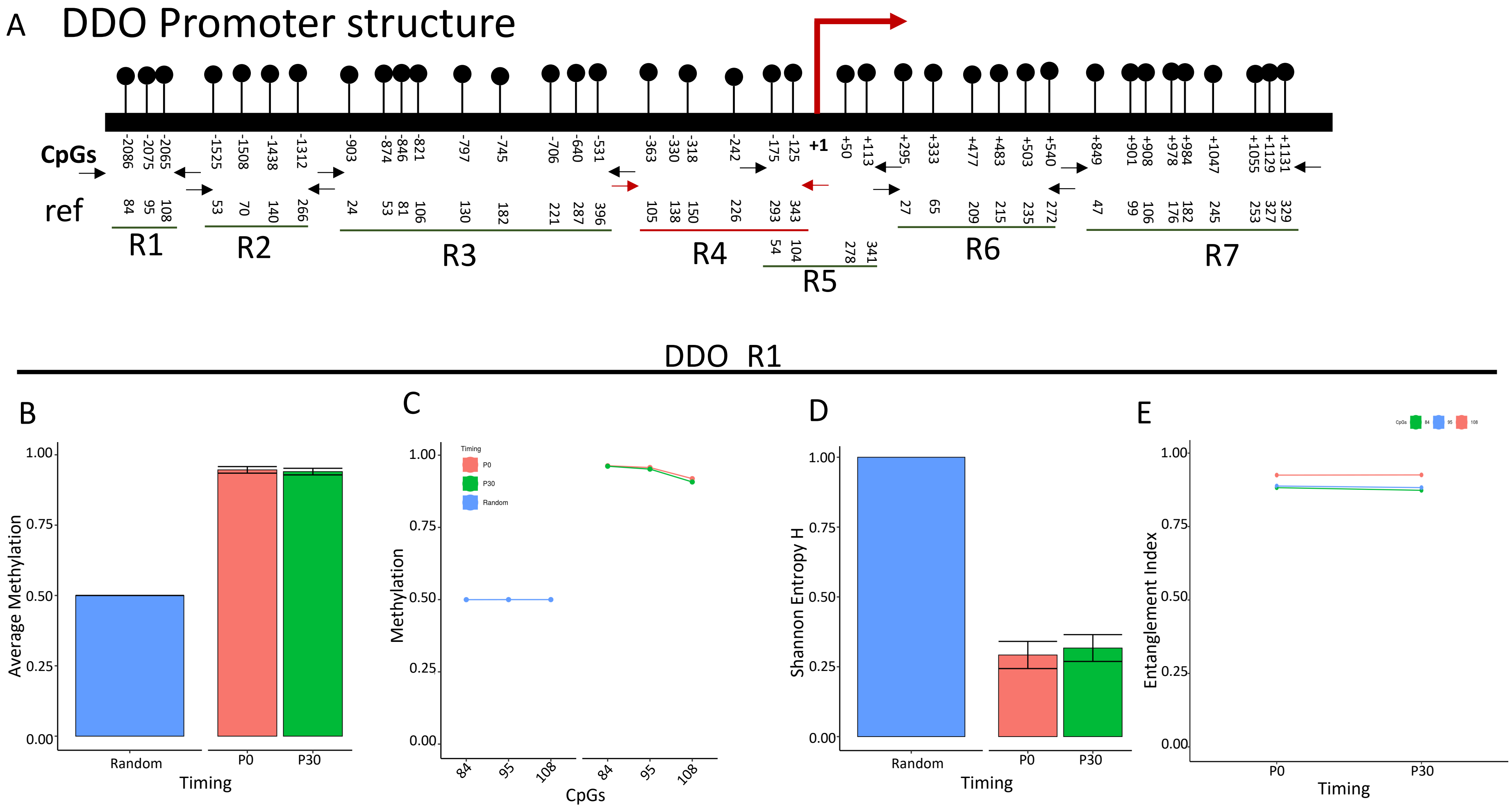

A DDO Promoter structure
CpGs
ref
R1
R2
R3
R4
R5
R6
R7
+1
DDO R1
B
Average Methylation
Random
P0
P30
Timing
C
Methylation
Timing
P0
P30
Random
CpGs
D
Shannon Entropy H
Random
P0
P30
Timing
E
Entanglement Index
P0
P30
Timing

Fig. S4

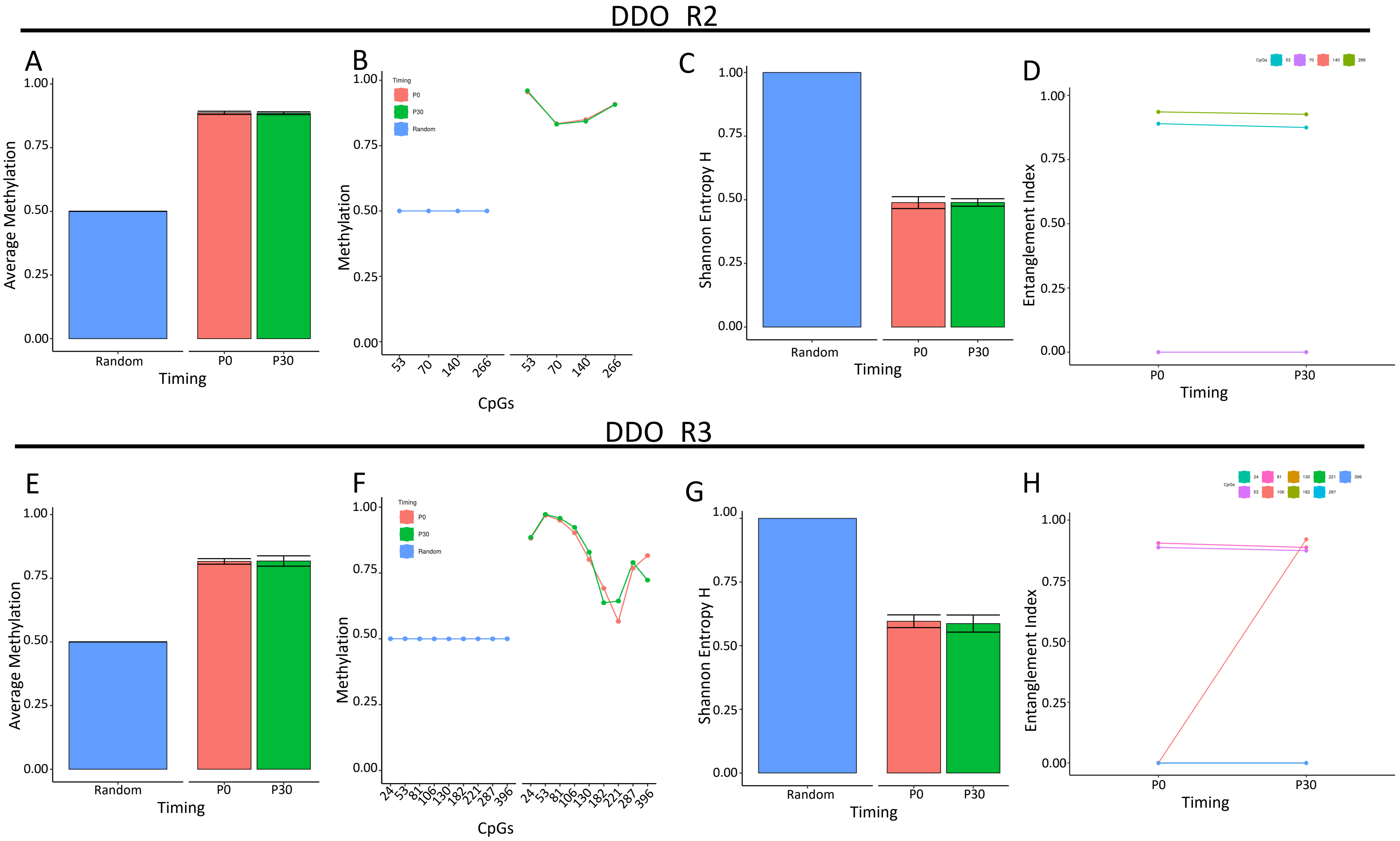
DDO R2
A
B
C
D
Average Methylation
Timing
Random
P0
P30
Methylation
CpGs
Timing
P0
P30
Random
Shannon Entropy H
Entanglement Index
DDO R3
E
F
G
H
Average Methylation
Methylation
Shannon Entropy H
Entanglement Index
Timing
CpGs
1.00
0.75
0.50
0.25
0.00
53
70
140
266
24
81
106
130
182
221
287
396

Fig. S5

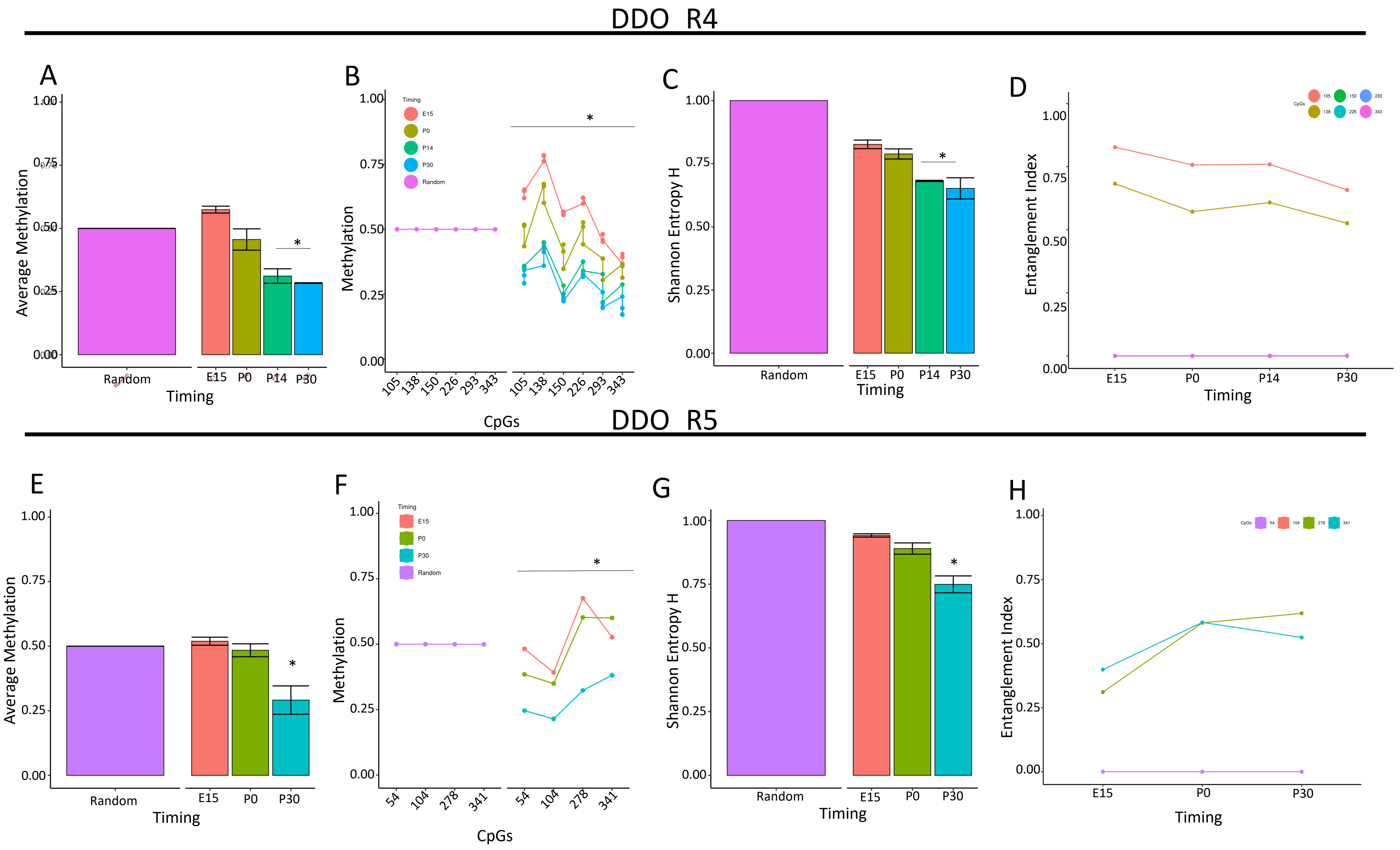
DDO R4
A
Average Methylation
1.00
0.75
0.50
0.25
0.00
Random
E15
P0
P14
P30
Timing
*
B
Methylation
Timing
E15
P0
P14
P30
Random
105
138
150
226
293
343
CpGs
*
C
Shannon Entropy H
Random
E15
P0
P14
P30
Timing
*
D
Entanglement Index
CpGs
105
138
150
226
293
343
E15
P0
P14
P30
Timing
DDO R5
E
Average Methylation
Random
E15
P0
P30
Timing
*
F
Methylation
Timing
E15
P0
P30
Random
54
104
278
341
CpGs
*
G
Shannon Entropy H
Random
E15
P0
P30
Timing
*
H
Entanglement Index
CpGs
54
104
278
341
E15
P0
P30
Timing

Fig. S6

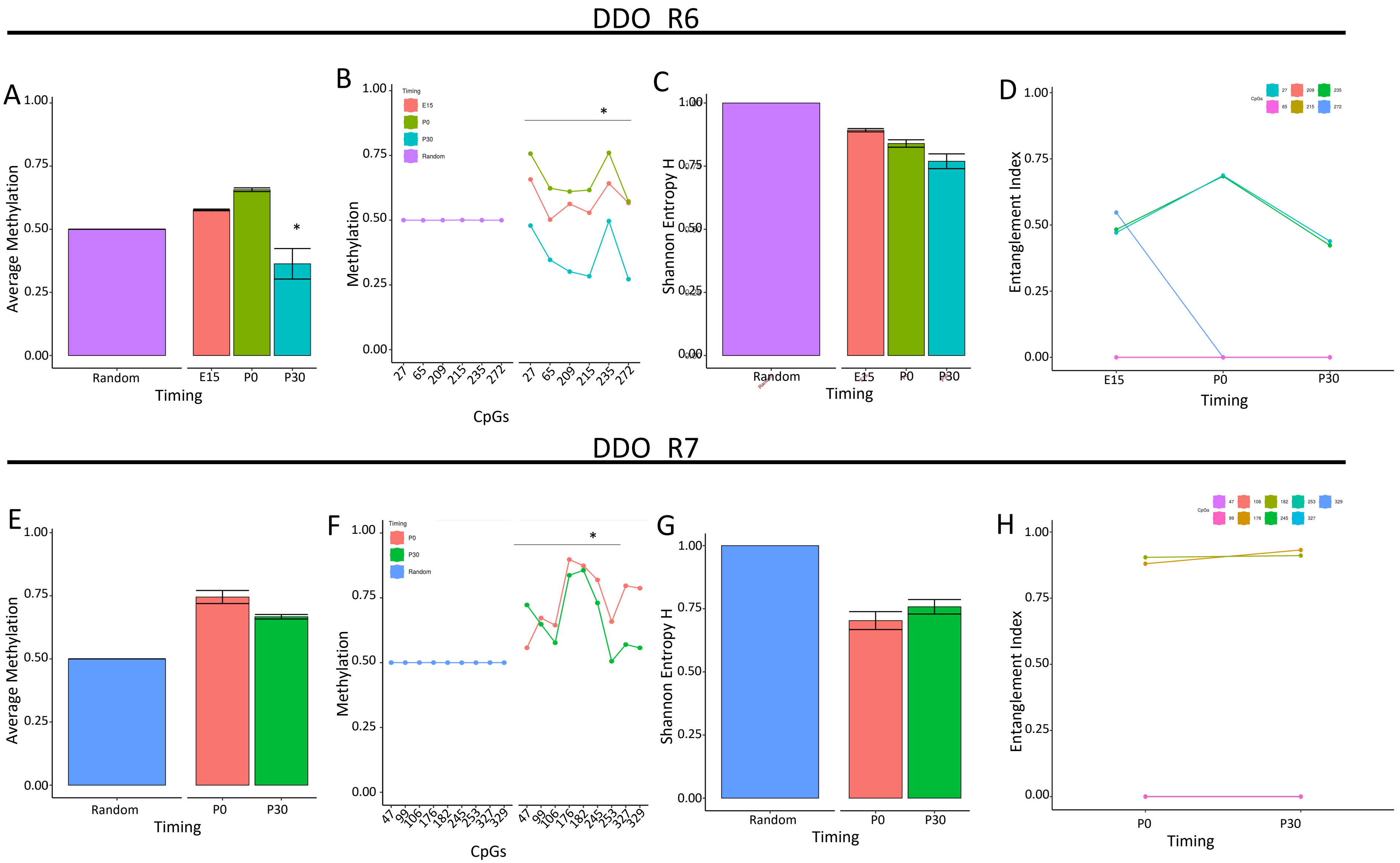
DDO_R6
A
Average Methylation
Random
E15
P0
P30
Timing
*
B
Methylation
Timing
E15
P0
P30
Random
*
27
65
209
215
235
272
CpGs
C
Shannon Entropy H
Random
E15
P0
P30
Timing
D
Entanglement Index
CpGs
27
209
235
65
215
272
E15
P0
P30
Timing
DDO_R7
E
Average Methylation
Random
P0
P30
Timing
F
Methylation
Timing
P0
P30
Random
*
47
99
106
176
182
245
253
327
329
CpGs
G
Shannon Entropy H
Random
P0
P30
Timing
H
Entanglement Index
CpGs
47
106
182
253
329
99
176
245
327
P0
P30
Timing

Fig. S7

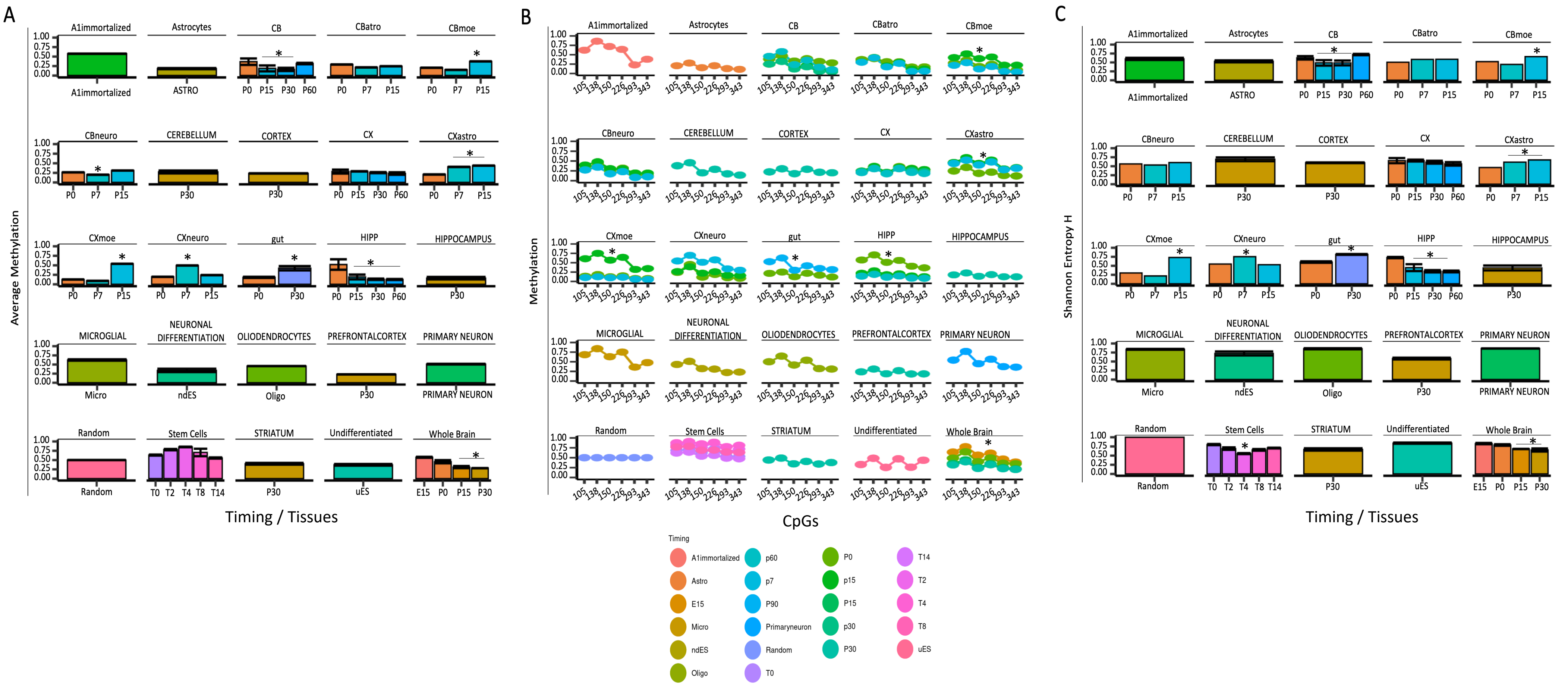
A
Average Methylation
A1immortalized
Astrocytes
CB
CBatro
CBmoe
CBneuro
CEREBELLUM
CORTEX
CX
CXastro
CXmoe
CXneuro
gut
HIPP
HIPPOCAMPUS
MICROGLIAL
NEURONAL DIFFERENTIATION
OLIODENDROCYTES
PREFRONTALCORTEX
PRIMARY NEURON
Random
Stem Cells
STRIATUM
Undifferentiated
Whole Brain
ASTRO
Micro
ndES
Oligo
uES
P0 P7 P15 P30 P60
E15
T0 T2 T4 T8 T14
Timing / Tissues
B
Methylation
105 138 150 226 293 343
CpGs
Timing
A1immortalized
Astro
E15
Micro
ndES
Oligo
p60
p7
P90
Primaryneuron
Random
T0
P0
p15
P15
p30
P30
T14
T2
T4
T8
uES
C
Shannon Entropy H
Timing / Tissues

Table S1

Analysis	Foward	Reverse	Gene
Bisulfite	5′-AAAGAATGGAAGAGGAAATTTAGA-3′	5′-TTTAGATAGGTTGATTTGGTTGGTG-3′	NANOG
Bisulfite	5′-AGAAAATAGTGATTTTAGAGTT-3′	5′-GTGTTGATGTTGTAGAGTTTGT-3′	TUBB3
Bisulfite	5′-GATAGGTAAGTAATTTATGGA-3′	5′-GATAATTGGGTATTATGTTA-3′	GFAP
Bisulfite	5′-GGATTGGAAGTGAAAATATTTAT-3′	5′-AGTTTTTGTTTAGATTAAATGGA-3′	BDNF plus
Bisulfite	5′-AATAACAAACTTAACTTCTATATA-3′	5′-GGTGATAGTTATTAAATTGAAG-3′	BDNF minus
Bisulfite	5′-TAAATATTTATAGAAGGGAATTGG-3′	5′-AAACAAACACTATAACATAAACC-3′	DDO
RNA	5′-CAAGGGTCTGCTACTGAGATGCTCTG-3′	5′-TTTTGTTTGGGACTGGTAGAAGAATCAG-3′	NANOG
RNA	5′-TTGGAACCTGGAACCATGGACAG-3′	5′-CTGTGCCCCCACCCAGTGA-3′	TUBB3
RNA	5′-GTCAGAAGGCCACCTCAAGAGG-3′	5′-TTGGGCAGCACACAGGCAGAA-3′	GFAP
RNA	5′-ACCCCTCACCCTAAATCCTG-3′	5′-TGCACGAATTACCAGAATCAG-3′	BDNF
RNA	5′-ACCACCAGTAATGTAGCGGC-3′	5′-GGTACCGGGGTATCTGCAC-3′	DDO
RNA	5′-TTCACCACCATGGAGAAGGCT-3′	5′—ACAGCCTTGGCAGCGCCAGT3′	GAPDH
